# Treatment of Emergencies

**Published:** 1887-06-11

**Authors:** 


### TILL THE DOCTOR COMES.

[Inquiries and Communications for this column should be addressed "Physician," care of the Editor of THE HOSPITAL.]

### XX.—TREATMENT OF EMERGENCIES.

*(Continued.)*

*Fractures and Dislocations.*—Great care is needed in these cases. As long as the skin is sound and the fracture is what is known as a simple fracture, good union may be expected ; but directly the skin is broken and air gets access to the broken end, the fracture becomes converted into a compound fracture, and the course is very much more severe. It is hardly necessary to point out how easily this misfortune may be brought about by want of care in the removal of the patient from the place of accident to his home. Many cases have occurred where the bone has been thrust through the skin by rough handling during the transit. In the case of the *leg* or *thigh*, the limb may be found shortened and perhaps twisted in some way. If so, take the foot firmly, replace it in its natural position, and draw gently downwards so as to bring the two limbs as nearly as possible to an equal length, but without applying force; then tie the two legs together with a scarf or handkerchief, just above the ankle, and again below and above the knee. In dislocations of the *hip* it will be found that no drawing down makes any difference to the length, or it may even be longer than the other from the first: here the only thing to be done is to tie the two legs together as before. In a fracture of the *arm* between the shoulder and elbow, let the hand lie loosely in an ordinary sling, and tie a scarf or handkerchief round the body so as to keep the arm close to the side. If the *forearm* is broken—*i.e.*, the part between the elbow and wrist-joints—let the whole forearm lie evenly in a sling, so adjusted as to support the whole length: in these fractures temporary splints may easily be applied. If the *shoulder* is dislocated, the arm will not come to the side, and the hand must simply be supported in a sling. It is not wise to try and reduce any dislocations without medical advice, for there may be a fracture as well, or some other injury, and permanent harm may be done to the limb. The only doubtful exceptions are the elbow and fingers. The *elbow* assumes often, when dislocated, such an inconvenient position, and usually slips in place so easily, that an attempt may be made to reduce it by steadying the upper arm, taking firm hold of the wrist, drawing it downwards, placing the knee in the bend of the elbow, and gradually bending the lower arm over it towards the shoulder. The *wrist* is seldom dislocated, in fact, such an injury may be left out of the question here. Dislocations of the *fingers* may generally be reduced by pulling strongly on them. Be careful about dislocations of the *thumb;* it is sometimes quite impossible to reduce them by any means. In fracture of the *collar-bone*, put the hand in a sling, and take the weight of the arm off the shoulder by a handkerchief passed round the elbow, and tied tightly over the opposite shoulder. In fractures of the *ribs*, bandage the chest firmly, or pin a flannel band firmly round it. This will limit the movements of the chest, and give great relief. Little can be done in other fractures, save taking care that no harm is done by unnecessary jolting or other means. In many of the above fractures, especially if the patients have to be carried some distance, it will be advisable to take extra precautions to prevent injury, by applying temporary splints. These are best made of thick pasteboard—a bonnet-box, or something of that kind: if the accident occurs out of doors, some bark, narrow bundles of straw, thin branches tied together, or thickly folded brown paper, may all be utilised to keep the limb temporarily at rest. These splints are best applied one on each side of the fractured limb, and kept in place by handkerchiefs. A patient with a fractured leg or thigh should be placed on a mattress, not feather-bed; and it will be all the better if a board is placed under this, to keep the splints from sinking down, and so disarrange the position of the limb. A bedpull—*i.e.*, a cross-bar of thick wood with a strong cord fixed to it, and this again made fast to a ring in the ceiling—will be of immense assistance to a patient in these cases; in fact, it is almost a necessity, and can easily be applied by any carpenter. What is known as a cradle, to keep the bedclothes off the limb, is also necessary; a bonnet-box will answer the purpose admirably. In lifting a fracture always have plenty of assistance. Let one take the limb at the bottom, and the other at the top of the splint, passing the fingers of each hand under the splint, and letting the thumbs meet, or as nearly so as possible, over the limb. In unwinding the bandage also, do not let the end lie loose, but gather it up closely as you proceed. A bandage may thus be removed in a very much shorter time than is otherwise necessary.

*(To be continued.)*

